# The semi-aquatic pondweed bugs of a Cretaceous swamp

**DOI:** 10.7717/peerj.3760

**Published:** 2017-09-05

**Authors:** Alba Sánchez-García, André Nel, Antonio Arillo, Mónica M. Solórzano Kraemer

**Affiliations:** 1 Departament de Dinàmica de la Terra i de l’Oceà and Institut de Recerca de la Biodiversitat (IRBio), Facultat de Ciències de la Terra, Universitat de Barcelona, Barcelona, Spain; 2 Institut de Systématique, Évolution, Biodiversité, Museum national d’Histoire naturelle, Paris, France; 3 Departamento de Zoología y Antropología Física, Facultad de Biología, Universidad Complutense de Madrid, Madrid, Spain; 4 Paläontologie und Historische Geologie, Sektion Paläozoologie I, Senckenberg Forschungsinstitut und Naturmuseum, Frankfurt am Main, Germany

**Keywords:** Heteroptera, Infrared microscopy, Litter amber, Paleoecology, Paleoethology, Spain

## Abstract

Pondweed bugs (Hemiptera: Mesoveliidae), considered a sister group to all other Gerromorpha, are exceedingly rare as fossils. Therefore, each new discovery of a fossil mesoveliid is of high interest, giving new insight into their early evolutionary history and diversity and enabling the testing of their proposed relationships. Here, we report the discovery of new mesoveliid material from Spanish Lower Cretaceous (Albian) amber, which is the first such find in Spanish amber. To date, fossil records of this family only include one species from French Kimmeridgian as compression fossils, two species in French amber (Albian-Cenomanian boundary), and one in Dominican amber (Miocene). The discovery of two males and one female described and figured as *Glaesivelia pulcherrima* Sánchez-García & Solórzano Kraemer gen. et sp. n., and a single female described and figured as *Iberovelia quisquilia* Sánchez-García & Nel, gen. et sp. n., reveals novel combinations of traits related to some genera currently in the subfamily Mesoveliinae. Brief comments about challenges facing the study of fossil mesoveliids are provided, showing the necessity for a revision of the existing phylogenetic hypotheses. Some of the specimens were studied using infrared microscopy, a promising alternative to the systematic study of organisms preserved in amber that cannot be clearly visualised. The new taxa significantly expand the fossil record of the family and shed new light on its palaeoecology. The fossils indicate that Mesoveliidae were certainly diverse by the Cretaceous and that numerous tiny cryptic species living in humid terrestrial to marginal aquatic habitats remain to be discovered. Furthermore, the finding of several specimens as syninclusions suggests aggregative behaviour, thereby representing the earliest documented evidence of such ethology.

## Introduction

Semi-aquatic bugs (Heteroptera, Gerromorpha) are the most successful group of insects inhabiting the water surface in a wide range of habitats, including the open ocean (Andersen, 1979; Wang et al., 2016). Gerromorpha consist of more than two thousand extant species worldwide and are composed of eight families: Mesoveliidae, Hebridae, Paraphrynoveliidae, Macroveliidae, Hydrometridae, Hermatobatidae, Veliidae, and Gerridae (Damgaard et al., 2012).

Mesoveliidae, so called ‘water treaders’ or ‘pondweed bugs’, are considered a sister group to all other families of the infraorder Gerromorpha (Andersen, 1982; Damgaard, 2008a; Damgaard, 2008b). The family has only 46 extant species in 12 genera (Andersen & Weir, 2004; Damgaard et al., 2012), but inhabits various types of humid terrestrial (hygropetric) to marginal aquatic and aquatic habitats (Andersen, 1982). All species prey on small arthropods. The eggs, which are the overwintering stage, are inserted into plant tissues, and there are five (rarely four) nymphal instars. Most species show wing dimorphism, but winged (macropterous) adults are usually uncommon (Andersen & Weir, 2004). According to Andersen (1982), the family contains the subfamilies Madeoveliinae and Mesoveliinae, although the monophyly of Mesoveliinae and its largest and most cosmopolitan genus, *Mesovelia* Mulsant & Rey, 1852, has been recently questioned by Damgaard et al. (2012) based on molecular data.

**Figure 1:**
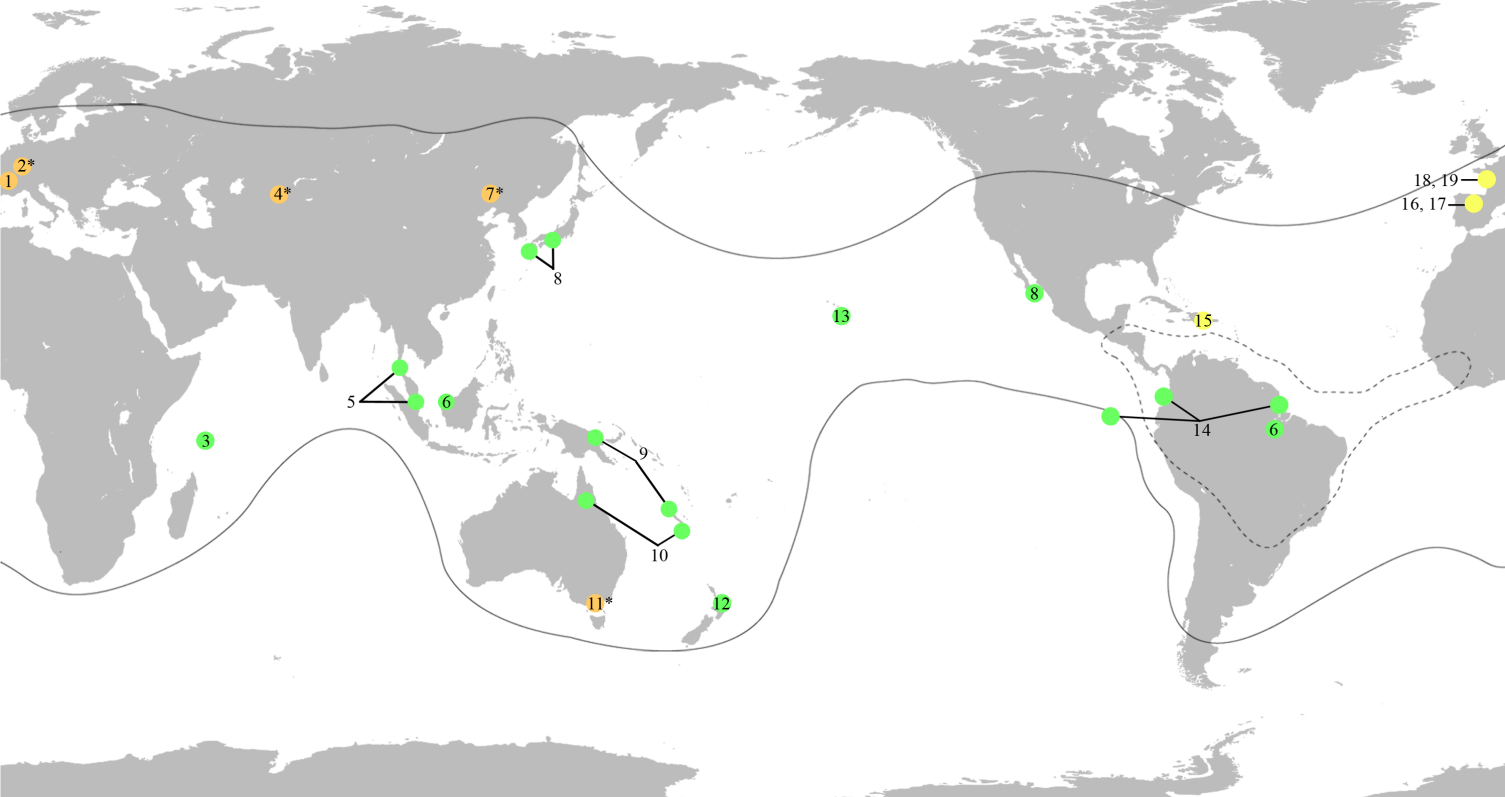
World map showing the distribution of the fossil and extant genera of Mesoveliidae, modified from Andersen & Polhemus (2003). Amber fossils are represented by yellow circles, compression fossils by orange circles and extant genera by green circles. Fossil taxa with controversial affinities are indicated by a superscript asterisk. Distribution of extant genera is based on information from Andersen & Polhemus (2003). Black lines delimit the distribution of the genus *Mesovelia*. Broken black lines delimit the distribution of the genera *Madeovelia* and *Mesoveloidea*. (1) *Gallomesovelia grioti*, Upper Jurassic (Upper Kimmeridgian) marine limestones from the area around Orbagnoux, Rhône Valley, France (Nel et al., 2014). (2) *Engynabis tenuis* Bode, 1953, Lower Jurassic Posidonia Shales, Germany, assigned to Eonabidae (Bode, 1953), related to *Karanabis* (Popov & Wootton, 1977), assigned to Mesoveliidae (Popov & Bechly, 2007), with unwarranted assignment to Mesoveliidae or even Gerromorpha (Andersen, 1982; Damgaard, 2008a; Yao, Zhang & Ren, 2012). (3) *Seychellovelia*. (4) *Karanabis kiritschenkoi* Bekker-Migdisova, 1962, Upper Jurassic Karabastau beds of Karatau, Kazakhstan, first assigned to Nabidae (Bekker-Migdisova, 1962), later to Gerridae (Popov, 1968), then to Mesoveliidae (Popov & Bechly, 2007), and now considered to be of uncertain taxonomic status (Yao, Zhang & Ren, 2012). (5) *Nereivelia*. (6) *Cryptovelia*. (7) *Sinovelia mega* Yao, Zhang & Ren, 2012 and *S. popovi* Yao, Zhang & Ren, 2012, Lower Cretaceous Yixian Formation in Huangbanjigou Chaomidian Village, Beipiao City, Liaoning Province, China, first assigned to Mesoveliinae (Yao, Zhang & Ren, 2012), but now considered Heteroptera *incertae sedis* (Damgaard et al., 2012). (8) *Speovelia*. (9) *Phrynovelia*. (10) *Austrovelia*. (11) *Duncanovelia extensa* Jell & Duncan, 1986, Lower Cretaceous Koonwarra Fossil Bed (Strzelecki Group) in Victoria, Australia, first assigned to Mesoveliidae (Jell & Duncan, 1986; Andersen, 1998; Yao, Zhang & Ren, 2012), but now considered Heteroptera *incertae sedis* (Damgaard et al., 2012). (12) *Mniovelia*. (13) *Cavaticovelia*. (14) *Darwinivelia*. (15) *Mesovelia dominicana*, Cenozoic (Middle Miocene) amber of La Toca mine, Dominican Republic (Garrouste & Nel, 2010). (16) *Iberovelia quisquilia* gen. et sp. n., Lower Cretaceous (Upper Albian) amber of Peñacerrada I, Spain. (17) *Glaesivelia pulcherrima* gen. et sp. n., Lower Cretaceous (Upper Albian) amber of Peñacerrada I, Spain. (18) *Emilianovelia audax*, Cretaceous (Albian–Cenomanian boundary) amber of Charentes, France (Solórzano Kraemer et al., 2014). (19) *Malenavelia videris*, Cretaceous (Albian–Cenomanian boundary) amber of Charentes, France (Solórzano Kraemer et al., 2014).

Probably due to the specific habitats they occupied, geological records of mesoveliids are exceedingly scarce, leaving many unanswered questions regarding their diversification over time. At present, only four fossil species have been assigned to the family with certainty, the Jurassic madeoveliine *Gallomesovelia grioti* Nel et al., 2014 (Late Jurassic from Orbagnoux, Rhône Valley, France, *c*.152 Ma), the two Cretaceous mesoveliine *Emilianovelia audax* Solórzano Kraemer & Perrichot, 2014 and *Malenavelia videris* Solórzano Kraemer & Perrichot, 2014 (Albian-Cenomanian amber from Charentes, France, *c*.100 Ma), and *Mesovelia dominicana* Garrouste & Nel, 2010 (middle Miocene Dominican amber, *c*.16 Ma) (Garrouste & Nel, 2010; Nel et al., 2014; Solórzano Kraemer et al., 2014). Damgaard et al. (2012) provided a comprehensive review of the known fossil records and discussed several misidentified taxa with equivocal relationships assigned to the family, including some of the oldest assigned to Gerromorpha (Fig. 1). According to phylogenetic-based estimations of divergence times, the aquatic and semi-aquatic true bugs Gerromorpha, Nepomorpha and Leptopodomorpha most likely originated successively from the Late Permian to Early Triassic (269–246 Ma) (Wang et al., 2016). However, the absence of Gerromorpha records from the Triassic and earlier is noteworthy given that Nepomorpha has been dated back to the Late Triassic and Leptopodomorpha to the Triassic/Jurassic boundary (Shcherbakov & Popov, 2002; Grimaldi & Engel, 2005), all of them inhabiting similar environments and possibly having similar taphonomic biases.

Here, we report new records of Early Cretaceous mesoveliids which are of significance for the palaeodiversity and palaeobiogeographical distribution of the family and comprise two new genera and species found in amber from Peñacerrada, Spain. This material represents the first record of the family in Cretaceous Spanish amber and is the earliest record of the subfamily Mesoveliinae. In addition, the presence of several individuals in the same piece of amber represents the earliest evidence of aggregative behaviour for Mesoveliidae.

Although Spanish amber samples are generally translucent, the darkened cuticle of the specimens, the occurrence of debris within some pieces, white foam (due to microbubbles), flow lines, and/or internal cracks greatly hamper the analysis of the morphological details of the inclusions. Therefore, infrared microscopy was used to visualise important details of the anatomy of the specimens more clearly. This technique is still not commonly used in research on amber inclusions, but some studies have shown its suitability in analysing detail-rich inclusions that are not visible with conventional optic methods (Riquelme et al., 2014; Huang et al., 2016).

## Materials and Methods

The present study is based on four fossil Mesoveliidae occurring in Lower Cretaceous amber from Peñacerrada. The deposits of Peñacerrada I and II (Álava amber), dated to Upper Albian (105 Ma: Barrón et al., 2015), are located in the northern slope of Sierra de Cantabria, in the southern limit of the Basque-Cantabrian Basin (northern Spain) (Alonso et al., 2000; Delclòs et al., 2007; Peñalver & Delclòs, 2010). Amber occurs in lutitic layers of deltaic origin with abundant coal. The arthropods found in it are usually hexapods, with arachnids occurring less frequently (Peñalver & Delclòs, 2010). Several crustacean specimens have also been reported in this amber (Sánchez-García et al., 2015). The families belonging to the order Hemiptera that have been found include Anthocoridae, Aradidae, †Hispanocaderidae, Hydrometridae, Mesoveliidae, Saldidae, †Tajmyraphididae, and Thaumastocoridae, although only †Tajmyraphididae (Peñalver & Wegierek, 2008), †Hispanocaderidae (Golub, Popov & Arillo, 2012), and Hydrometridae (Sánchez-García, Arillo & Nel, 2016) have been studied.

Three of the mesoveliids described in the present study (MCNA numbers 12804, 12805, and 12806) were discovered as syninclusions in a large piece of amber. This piece was trimmed into several smaller pieces to enable better examination of individual inclusions. Specimen MCNA 13326 was found isolated. Amber pieces were polished and embedded in synthetic resin (EPO-TEK 301), as described in Nascimbene & Silverstein (2000). Although the weathered opaque surface of the amber was removed, the inclusions remained occluded with particles and bubbles. The material was studied under a Motic BA310 compound microscope and a Nikon SMZ1500 stereomicroscope, and measurements were obtained with Motic Images Plus 2.0 software on the Motic BA310 compound microscope. All measurements were recorded in millimeters. Colour photomicrographs were taken with an AmScope MU900 camera attached to the Nikon SMZ1500 stereomicroscope, using the AmScope ToupView 3.5 software (Muséum National d’Histoire Naturelle, Paris, France), as well as with a Moticam 2500 camera attached to the Motic BA310 compound microscope, using the Motic Images Plus 2.0 software (Universitat de Barcelona, Barcelona, Spain). Some of the original photographs were z-stacked using the Helicon Focus 3.10 software. Drawings were made using a Leica drawing tube attached to a Leica MZ12 stereomicroscope (Senckenberg Forschungsinstitut und Naturmuseum, Frankfurt, Germany). Infrared reflected photomicrographs were taken with a Nikon Eclipse ME600D at the Senckenberg Forschungsinstitut und Naturmuseum (see Brocke & Wilde (2001) for precise technical information). Original photographs were z-stacked using the Photoshop CS3 software.

We used the same morphological terminology as Andersen (1982) and followed Andersen (1999) for the systematic analysis. The material is housed in the Museo de Ciencias Naturales de Álava (MCNA), Vitoria-Gasteiz (Álava, Spain).

The electronic Portable Document Format (PDF) version of this article conforms to the requirements of the amended International Code of Zoological Nomenclature, and hence the new names contained herein are available under that Code from the electronic edition of this article. This published work and the nomenclatural acts it contains have been registered in ZooBank, the online registration system for the ICZN. The ZooBank Life Science Identifiers (LSIDs) can be resolved and the associated information viewed through any standard web browser by adding the LSID after http://zoobank.org/. The LSID for this publication is: (urn:lsid:zoobank.org:pub:5EBBDE3B-9A12-4476-BC96-776651350175). The online version of this work is archived and available on PeerJ, PubMed Central, and CLOCKSS.

## Results

### Systematic palaeontology

**Table:** 

Infraorder: Gerromorpha Popov, 1971
Family: Mesoveliidae Douglas & Scott, 1867

Genus *Iberovelia* Sánchez-García & Nel **gen. n**. urn:lsid:zoobank.org:act:9093AD2B-3AFC-4345-B35B-08139856ECFA

Type species *Iberovelia quisquilia* Sánchez-García & Nel **sp. n.**

**Etymology.** Derived from *ibero*-, in reference to the Iberian Peninsula from which the amber originates and -*velia*, a common suffix for Mesoveliidae genera.

**Diagnosis.** The genus is distinguished from all other Mesoveliidae genera by its unique combination of the following characters: small-sized apterous female form, length 1.6 mm. Head not deflected, extended in front of the eyes, slightly shorter than thorax, and clearly narrower than pronotum; anteclypeus with a pad of long erect hairs; eyes large; ocelli absent; antennae flagelliform, very long, clearly surpassing the abdominal apex, the first segment with two ante-apical spinous hairs; rostrum reaching metacoxae. Pronotum without collar and longer than mesonotum; metafemur long and clearly surpassing the abdominal apex; pro-, meso-, and metafemur with one, two, and one spinous hair, respectively; metatibia covered with scattered spinous hairs; first segment of tarsus the shortest, second segment shorter than third except in the metatarsus (second metatarsal segment almost twice the length of the third). Female genital segments only slightly protruding from pregenital abdomen; gonapophyses elongate and laciniate; gonoplacs small. Male unknown.

**Figure 2:**
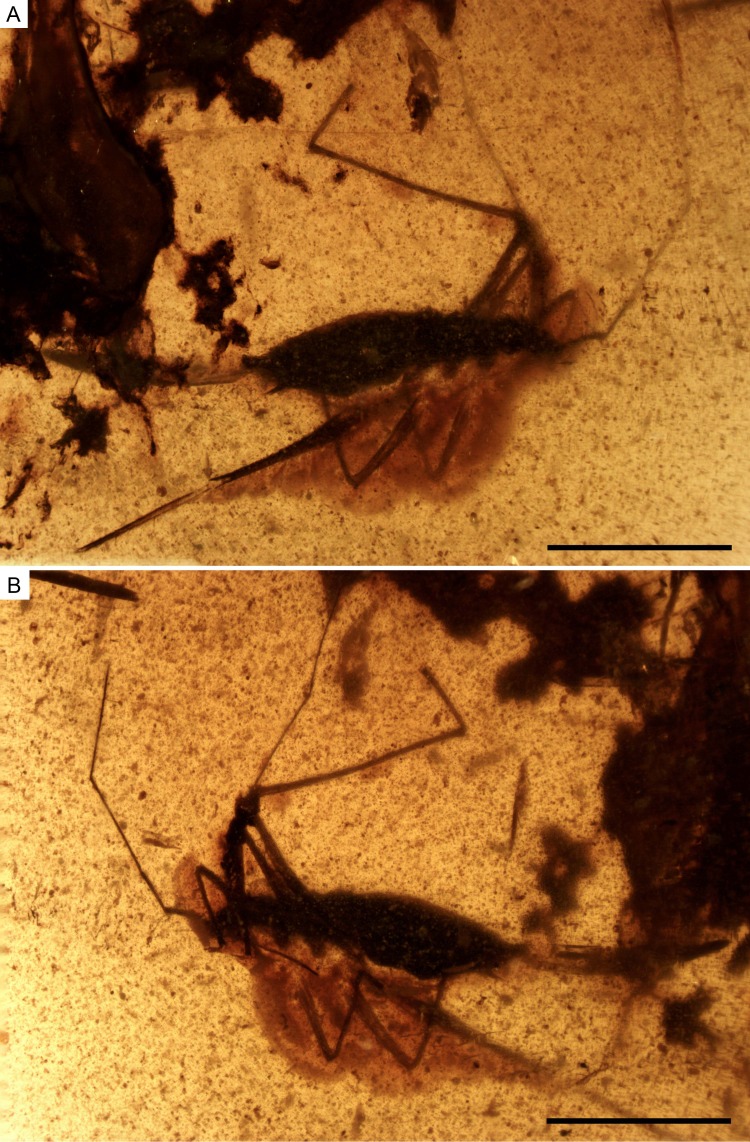
Photomicrographs of the holotype of *Iberovelia quisquilia* gen. et sp. n., female, MCNA 12804. (A) Dorso-lateral habitus. (B) Ventro-lateral habitus. Scale bars: 1 mm. Images combine consecutive photographs taken at successive focal planes.

**Figure 3:**
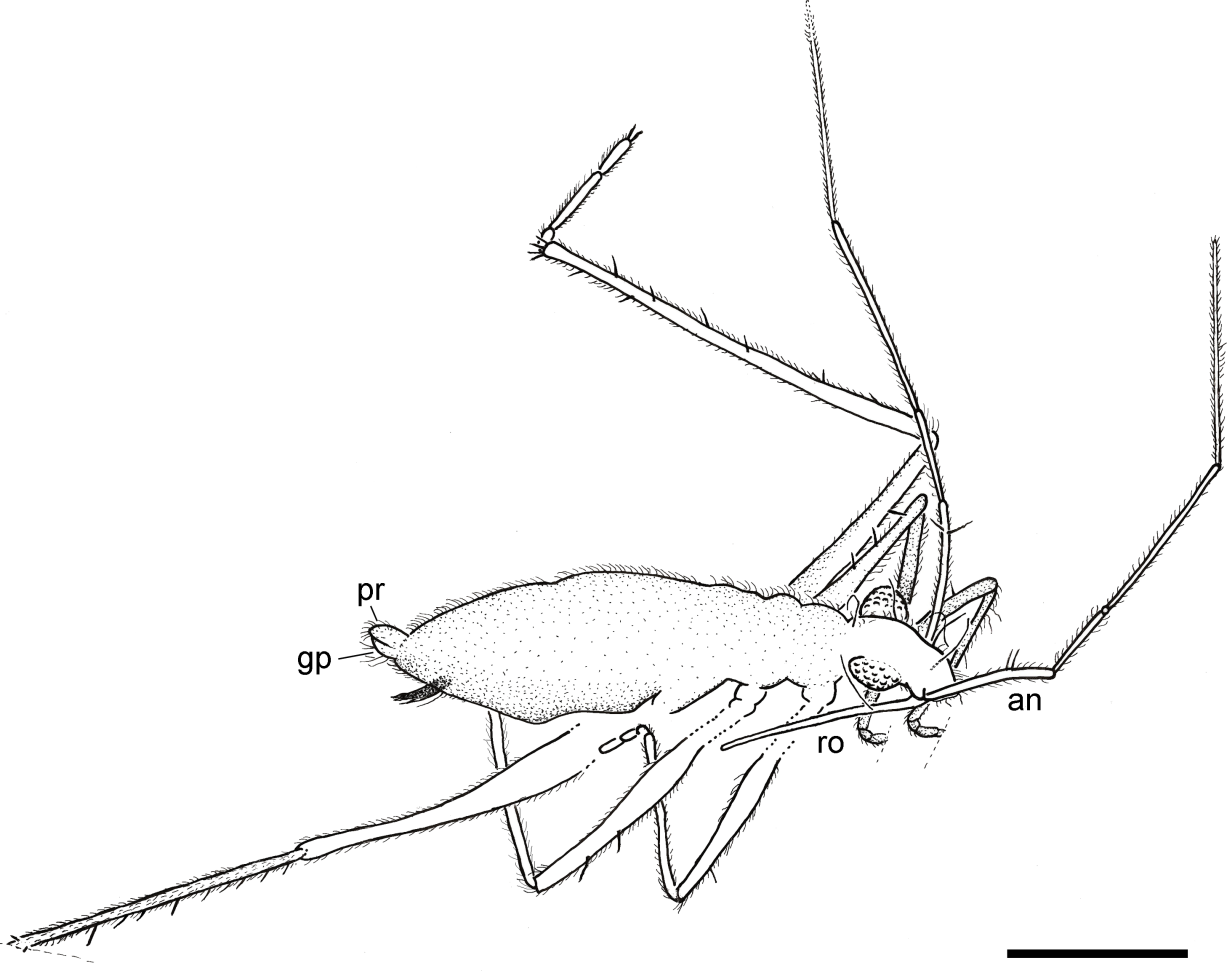
Camera lucida drawing of the holotype of *Iberovelia quisquilia* gen. et sp. n., female, MCNA 12804, in the dorso-lateral habitus. an, antenna; gp, gonoplacs; pr, proctiger; ro, rostrum. Scale bar: 0.5 mm.

**Figure 4:**
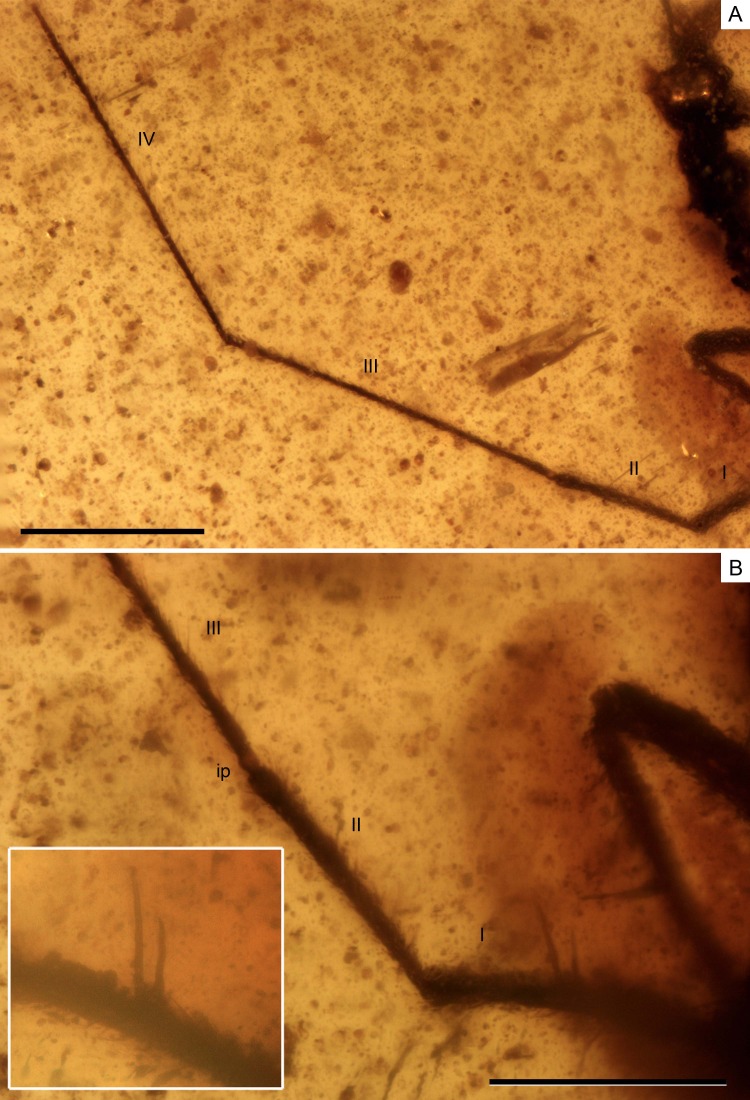
Photomicrographs of the holotype of *Iberovelia quisquilia* gen. et sp. n., female, MCNA 12804. (A) Antenna. (B) Detail of the first and second antennal segments, the inset showing a magnification of the two spinous hairs of the first antennal segment. I–IV, antennal segments I–IV; ip, internodial piece. Scale bars: (A) 0.3 mm, (B) 0.2 mm. Images combine consecutive photographs taken at successive focal planes.

**Figure 5:**
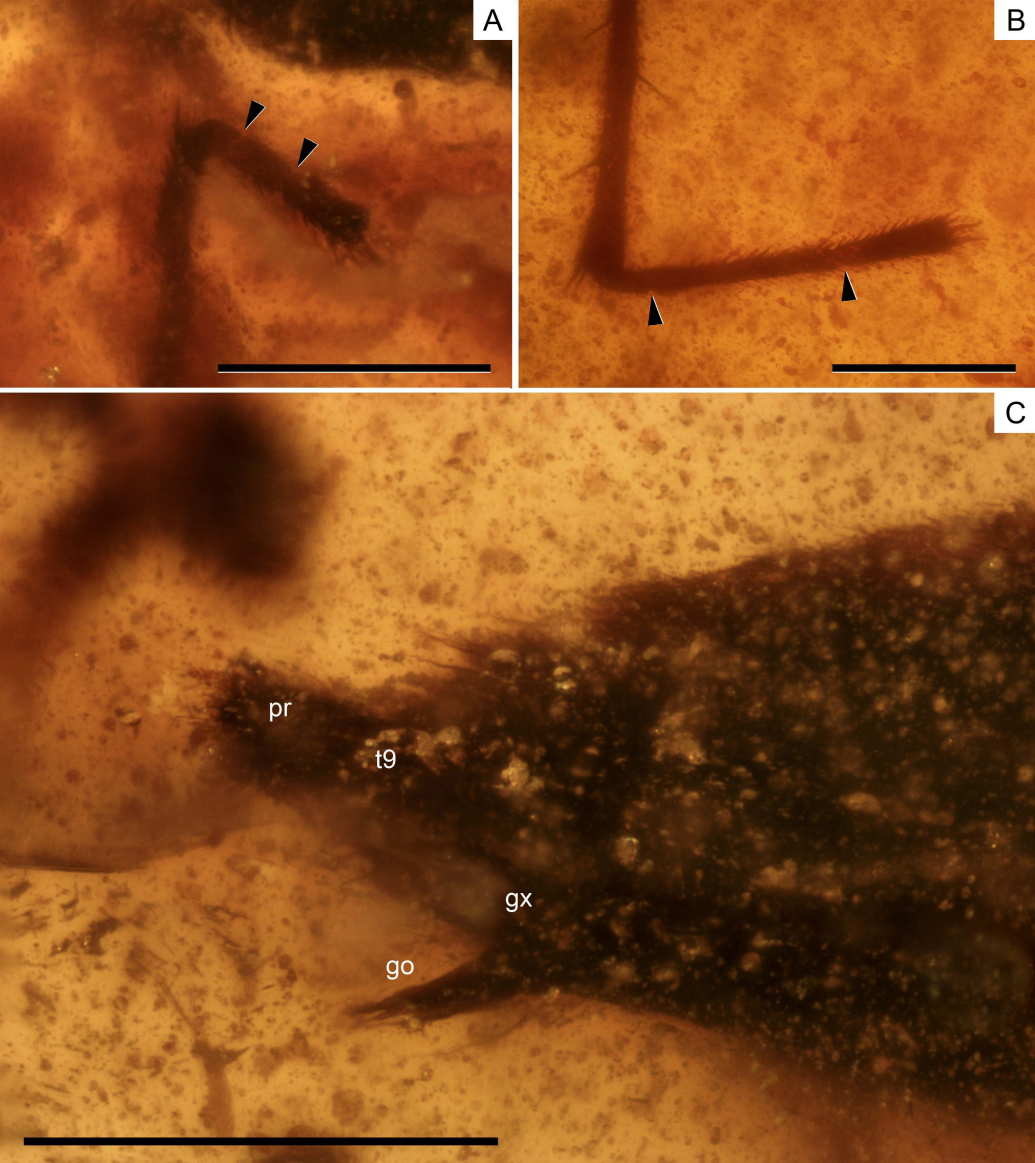
Photomicrographs of the holotype of *Iberovelia quisquilia* gen. et sp. n., female, MCNA 12804. (A) The right protarsus. (B) The left metatarsus. (C) The abdominal apex. Limits between tarsomeres are indicated with arrows. go, gonapophysis; gx, gonocoxa; pr, proctiger; t9, tergum 9. Scale bars: (A, B) 0.2 mm, (C) 0.3 mm. Images combine consecutive photographs taken at successive focal planes.

*Iberovelia quisquilia* Sánchez-García & Nel **sp. n.** urn:lsid:zoobank.org:pub:5EBBDE3B-9A12-4476-BC96-776651350175

(Figs. 2–5, Table 1)

**Table 1:** Characters for separation of the different genera of Mesoveliidae. Modified from Andersen & Polhemus (1980), Polhemus & Polhemus (1989) and Andersen & Polhemus (2003). The table was completed with characters described by Andersen (1982), Andersen (1999), Nel et al. (2014) and Solórzano Kraemer et al. (2014).

Characters	*Madeovelia* Poisson, 1959	*Mesoveloidea* Hungerford, 1929	†*Gallomesovelia* Nel et al., 2014	*Mesovelia* Mulsant & Rey, 1852	*Speovelia* Esaki, 1929	*Cavaticovelia* (Gagné & Howarth, 1975)	*Austrovelia* Malipatil & Monteith, 1983	*Cryptovelia* Andersen & Polhemus, 1980
	(1 sp.)	(2 ssp.)	(1 sp.)	(28 ssp.)	(3 ssp.)	(1 sp.)	(2 ssp.)	(2 ssp.)
Size, mm	?	3.8	6.0	2–4.5	2.6–4.0	3.6–4.2	0.9–1.5	1.1–1.3
Head length	Shorter than thorax	Shorter than thorax	Shorter than thorax	Shorter than thorax	Shorter than thorax	Subequal to or longer than thorax	Longer than thorax	Longer than thorax in *C. stysi* Subequal to (♀) or shorter (♂) in *C. terrestris*
Ventral head	Simple	Simple	?	Simple	Simple	Simple	Plate-like raised, distinctly carinate	Distinctly carinate
Eyes	Normal	Normal	Normal	Normal	Normal	Reduced	Normal or reduced	Strongly reduced
Antennae form	Subflagelliform	Subflagelliform	?	Subflagelliform and flagelliform	Subflagelliform	Subflagelliform	Flagelliform	Flagelliform
Antennae length	?	?	?	Very long	Very long	Longer than body	Shorter than body	Subequal to or shorter than body
Rostrum extending to	Meso- or metacoxae	Meso- or metacoxae	Mesocoxae	Meso- or metacoxae	Meso- or metacoxae	Abdomen	Metacoxae	Abdomen
Antennal spines	No	No	?	Yes	Yes	Yes	No	No, bristles
Femoral spines	Yes, weak	Yes, weak	?	Yes, strong	Yes, strong	Yes, weak	No	Yes/No
Mesonotum	?	?	?	Longer than pronotum	Longer than pronotum	Shorter than pronotum	Subequal to or shorter than pronotum	Subequal to pronotum
Posterior margin of metanotum	?	?	?	Straight	Curved	Straight	Curved	Straight
Metatarsal segments	1 < 2 < 3	1 < 2 ≈ 3 / 1 < 2 > 3	?	1 < 2 ≥3	1 < 2 > 3	1 < 2 > 3	1 < 2 < 3	1 < 2 < 3 in *C.terrestris* and ♀*C. stysi*; 1 < 2 > 3 in ♂*C. stysi*^a^
Wings	Monomorphic macropterous	Monomorphic macropterous	Macropterous	Dimorphic (macropterous and apterous)	Apterous	Apterous	Apterous	Apterous
Scent gland pore on tergite IV		Before middle	?	Before middle	Before middle	Behind middle	Behind middle	Behind middle
Gonoplacs	?	Large	?	Large	?	Large	Elongate	Small

Characters	*Darwinivelia* Andersen & Polhemus, 1980	*Mniovelia* Andersen & Polhemus, 1980	*Nereivelia* Polhemus & Polhemus, 1989	*Phrynovelia* Horváth, 1915	*Seychellovelia* Andersen & Polhemus, 2003	†*Emilianovelia* Solórzano Kraemer & Perrichot, 2014	†*Malenavelia*Solórzano Kraemer & Perrichot, 2014	†*Glaesivelia* gen. n.	†*Iberovelia* gen. n.
	(3 ssp.)	(1 sp.)	(2 ssp.)	(4 ssp.)	(1 sp.)	(1 sp.)	(1 sp.)	(1 sp.)	(1 sp.)
Size, mm	1.8	1.8–2.0	1.7–2.2	1.5–1.8	1.4–1.75	1.4–1.7	1.5–1.6	1.6	1.6
Head length	Longer than thorax	Shorter than thorax	Subequal to or longer than thorax	Longer than thorax	Subequal to or longer than thorax	Subequal to or longer than thorax	Subequal to or longer than thorax	Longer than thorax	Shorter than thorax
Ventral head	Plate-like raised, distinctly carinate	Weakly carinate	Plate-like raised, weakly carinate	Simple or weakly carinate	Weakly carinate	Simple	Distinctly carinate	Distinctly carinate	?
Eyes	Reduced	Very large	Normal	Normal	Normal	Normal	Normal	Normal	Normal
Antennae form	Subflagelliform	Flagelliform	Flagelliform	Flagelliform	Flagelliform	Flagelliform^b^	Flagelliform^b^	Flagelliform	Flagelliform
Antennae length	Shorter than body	Shorter than body	Subequal to or shorter than body	Shorter than body	Subequal to or shorter than body	Longer than body	Shorter than body	Shorter than body	Longer than body
Rostrum ending to	Meso- or metacoxae	Metacoxae	Abdomen	Meso- or metacoxae	Abdomen	Metacoxae	Metacoxae	Metacoxae	Metacoxae
Antennal spines	No	No, bristles	No	No	No, bristles	No, bristles	?	Yes	Yes
Femoral spines	Yes, weak	No	No	No	No	Yes, weak	No (apparently)	Yes, strong	Yes, strong
Mesonotum	Subequal to or shorter than pronotum	Subequal to or shorter than pronotum	Subequal to or shorter than pronotum	Subequal to or shorter than pronotum	Shorter than pronotum	Slightly shorter than pronotum	Shorter than pronotum	Shorter than pronotum	Shorter than pronotum
Posterior margin of metanotum	Curved	Curved	Straight or weakly curved	Straight	Curved	Weakly curved^c^	Weakly curved^c^	Weakly curved	?
Metatarsal segments	1 < 2 < 3	1 < 2 > 3	1 < 2 < 3	1 < 2 < 3	1 < 2 < 3	1 < 2 > 3	1 < 2 > 3	1 < 2 ≈ 3	1 < 2 > 3
Wings	Apterous	Apterous	Apterous	Apterous	Apterous	Apterous	Apterous	Apterous	Apterous
Scent gland pore on tergite IV	?	Behind middle	Before middle	Before middle	Before middle	Middle	?^d^	?	?
Gonoplacs	Large	Small	?	Small	Small	Large	Small	Small	Small

**Notes.**

a 1 Character state follows Andersen (1999). However, in the original description of C. *terrestris* the second and third metatarsal segments are described as subequal.

b Character modified from Solórzano Kraemer et al. (2014).

c The segment with a weakly curved posterior margin is the metanotum and not the mesonotum as was incorrectly described in its diagnosis.

d The scent gland pore is situated on tergite IV. According to figures in Solórzano Kraemer et al. (2014), the orifice described as a scent gland in tergite III of *Malenavelia* must be an artefact.

**Type material.** Holotype MCNA 12804, female, virtually complete, dorsolaterally and ventrolaterally exposed. Preserved in dark yellow turbid amber trimmed to 0.8 ×0.6 × 0.2 mm (in a trapezoid resin measuring 2.0 ×1.6 × 0.2 mm) and containing many impurities and bubbles. The amber is also darkened near the inclusion. The entire head, rostrum, and even antennae are preserved, as are the thorax and abdomen (including genitalia). Most of the left protarsus (missing from the third segment) and left mesotarsus (missing from the second segment) are lost at the surface of the amber, while the right mesotarsal segments are not distinguishable due to preservation. The right metathoracic leg is also missing below the distal third of the tibia. Ventral head details and thorax and abdomen segmentation cannot be assessed due to preservation. Syninclusions comprised the holotype and allotype of *Glaesivelia pulcherrima* (MCNA 12805 and MCNA 12806, see below) and one Diptera Dolichopodidae (*Microphorites* sp., MCNA 12807).

**Age and locality.** Lower Cretaceous (Upper Albian); Peñacerrada I amber site (Peñacerrada I = Moraza), eastern area of the Basque-Cantabrian Basin, Burgos, northern Spain.

**Etymology.** The specific epithet *quisquilia* is Latin for litter, and makes reference to the putative habitat (leaf litter in moist terrestrial environments) of the specimen.

**Diagnosis.** Same as for the genus (*vide supra*).

**Description of the holotype.** Female (Figs. 2–5). Apterous form. Body (Figs. 2 and 3) suboval and elongate, very small, length 1.64 mm, greatest width (across abdomen) 0.39 mm, length 4.20× the greatest width. Body surface and appendages covered with fine to coarse recumbent to semi-erect long setae.

Head (Fig. 3) relatively long, not deflected, clearly extended in front of the eyes, length 0.31 mm, much longer than wide, greatest width (across eyes) 0.15 mm, with sides nearly parallel; slightly shorter than thorax and clearly narrower than pronotum; anterior part slightly declivent in side view; ventral lobes and ventral head details not visible; anteclypeus with a pad of long erect setae. Three pairs of trichobothria on dorsal head surface, long, apparently not equally spaced in the longitudinal direction; one of the pairs occurs towards the base of the head, just before the posterior margin of the eyes, while the other two are inserted into the anterior part of the head, well in front of the eyes. No distinct median groove on head.

Compound eyes (Fig. 3) spherical, large, diameter 0.12 mm, that are not touching and only slightly separated from the anterior margin of the pronotum, with more than 30 ommatidia; ocular setae not visible. Ocelli absent (as typically occur in extant apterous forms).

Antennae (Fig. 4A) very long, length 1.81 mm, surpassing the length of the body when directed backwards, flagelliform, with segments 3–4 much longer and thinner than segments 1–2; antennal tubercles moderately prominent, slightly projected laterally, and situated near apex of head; first antennal segment longer than second, lengths 0.35 and 0.24 mm, respectively, the first segment with two ante-apical spinous hairs (Fig. 4B); third segment 0.59 mm long; fourth segment the longest, length 0.63 mm, uniformly sclerotised throughout.

Rostrum (Fig. 3) long and slender with apex reaching metacoxae.

Thorax (Fig. 3) not prolonged, length 0.36 mm, greatest width 0.29 mm; pronotum without collar; pro-, meso-, and metanotum only visible in side view, thus impeding measurements; pronotum longer than mesonotum; intersegmental sutures poorly visible, the posterior margin of metanotum apparently curved. Metathoracic scent gland not visible.

Legs (Fig. 3) inserted close to the ventral midline of the body, long, rather slender, and with strong spinous hairs; metathoracic leg the longest; coxae relatively long; femora moderately incrassate in the middle, nearly twice as thick as the corresponding tibiae; metafemur the longest, clearly surpassing the abdominal apex; anterior margin of pro-, meso-, and metafemur with at least one, two, and one ante-apical spinous hair, respectively; tibiae slender; metatibia the longest and covered with scattered spinous hairs, viz., with only two spinous hairs on the inner margin and a series of spinous hairs on the outer margin, plus up to four spinous hairs distally; lengths of profemur and protibia: 0.49 and 0.50 mm; mesofemur and mesotibia: 0.64 and 0.55 mm; metafemur and metatibia: 0.99 and 1.21 mm; tarsi with three segments; tarsal segments increasing in length from first to third segment (Fig. 5A) except in the metatarsus (Fig. 5B) (second metatarsal segment almost twice the length of the third); basitarsomere of all legs very short and subcylindrical; lengths of protarsal segments 1–3: 0.03, 0.05, and 0.07 mm; length of mesotarsal segment 1: 0.03 mm; lengths of metatarsal segments 1–3: 0.05, 0.21, and 0.12 mm; claws simple, slender, and inserted apically into the distal tarsal segment; arolia not visible.

Abdomen (Fig. 3) long, length 0.97 mm, greatest width 0.39 mm, clearly widened at the middle, with broadly concave sides; mediotergites tapering in width towards the abdominal apex. Abdominal scent gland not visible. Genital segments (Fig. 5C) large and only slightly protruding from pregenital abdomen; gonocoxae large and plate-shaped; gonapophyses elongate and laciniate, slightly sclerotised apically; tergum 9 relatively small; proctiger cone-shaped; gonoplacs small.

Male unknown.
Macropterous adult form unknown.

**Observational notes.** Direct sexual determination of the holotype was possible given the preservation of the female genital segments composed of gonocoxae, gonapophyses, gonoplacs, and the cone-shaped proctiger in the distal part of the abdomen. The gonoplacs are primitively large in mesoveliids, but have become smaller in some taxa such as *Cryptovelia*, *Mniovelia*, and *Phrynovelia*. Furthermore, there are differences in the ovipositor serration (from slightly to distinctly serrated), although this could not be assessed in *Iberovelia* due to preservation. It should be noted that Recent mesoveliids have a well-developed ovipositor that is functionally associated with the unique egg structure of mesoveliids and their habit of embedding eggs into living or dead plant tissues or narrow crevices and holes in the soil litter layer, instead of placing them on top of the substrate (Andersen, 1982).

Genus *Glaesivelia* Sánchez-García & Solórzano Kraemer **gen. n.** urn:lsid:zoobank.org:act: DAC68787-5F0A-462A-BCBB-2CDD1E6991D6

Type species *Glaesivelia pulcherrima* Sánchez-García & Solórzano Kraemer **sp. n.**

**Etymology.** Derived from Latin *glaesum* to mean ‘of amber’ and -*velia*, a common suffix for Mesoveliidae genera.

**Diagnosis.** The genus is distinguished from all other Mesoveliidae genera by its unique combination of the following characters: small-sized apterous form, length 1.6 mm. Head not deflected, extended in front of the eyes, subequal in length to the middorsal length of thorax in the female, but longer than thorax in the male, clearly narrower than pronotum; anteclypeus with a pad of long erect hairs; ventral surface of head laterally bordered by ridged longitudinal carina; eyes large; ocelli absent; antennae flagelliform, long, not reaching the abdominal apex, the first segment with three ante-apical spinous hairs in the female and two ante-apical spinous hairs in the male; rostrum reaching metacoxae. Pronotum without collar and longer than mesonotum, the posterior margin slightly pointed medially; metanotum with the posterior margin weakly curved; metafemur short and not surpassing the abdominal apex; pro-, meso-, and metafemur with one, two, and two spinous hairs, respectively, in the female but without spinous hairs in the male; metatibia covered with scattered spinous hairs in the female and with an inner row of spinous hairs in the male; first segment of tarsus the shortest, second segment shorter than third except in the metatarsus (second metatarsal segment subequal in length to the third). Female genital segments clearly protruding from pregenital abdomen; gonapophyses elongate and laciniate; gonoplacs small.

**Figure 6:**
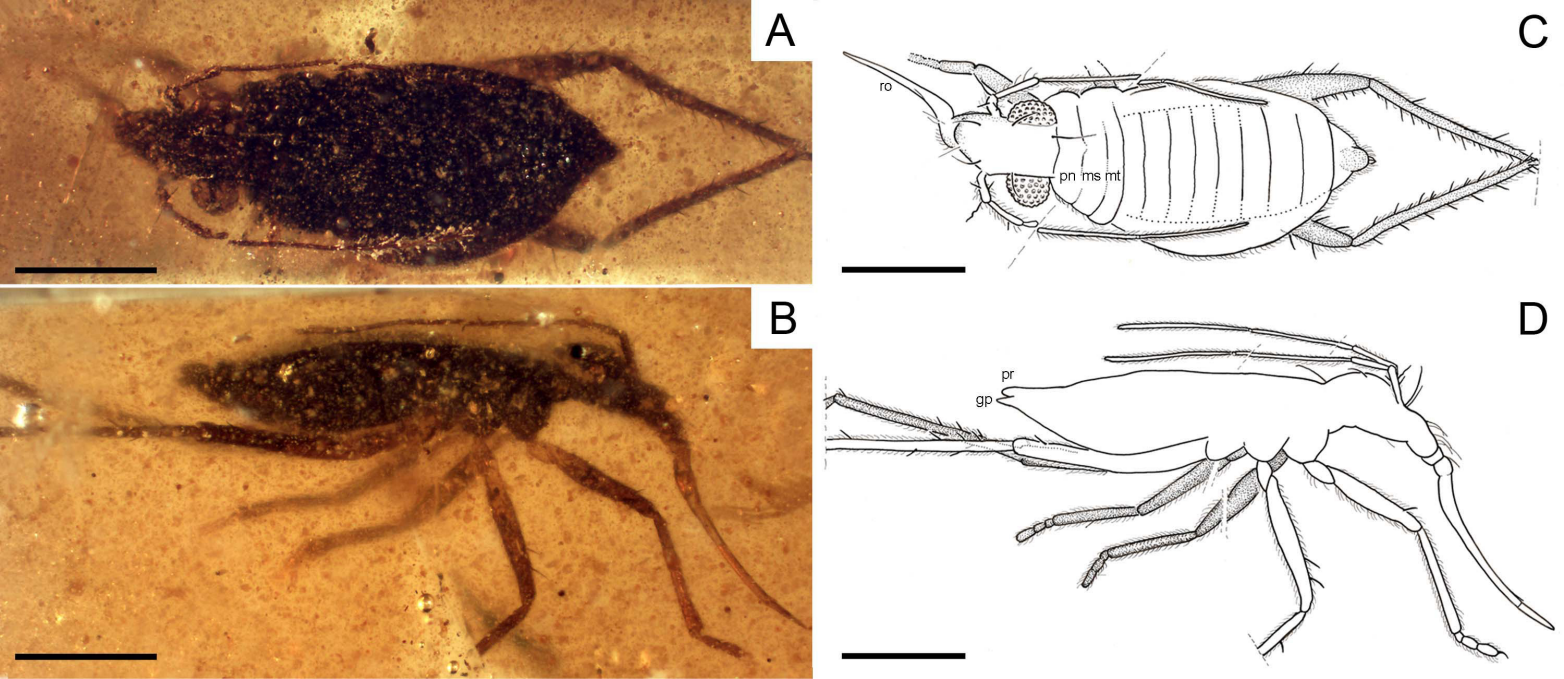
Photomicrographs and camera lucida drawings of the holotype of *Glaesivelia pulcherrima* gen. et sp. n., female, MCNA 12806. (A) Dorsal habitus. (B) Lateral habitus. (C) Drawing from A. (D) Drawing from B. gp, gonoplacs; mt, metanotum; ms, mesonotum; pn, pronotum; pr, proctiger; ro, rostrum. Scale bars: 0.5 mm. Images combine consecutive photographs taken at successive focal planes.

**Figure 7:**
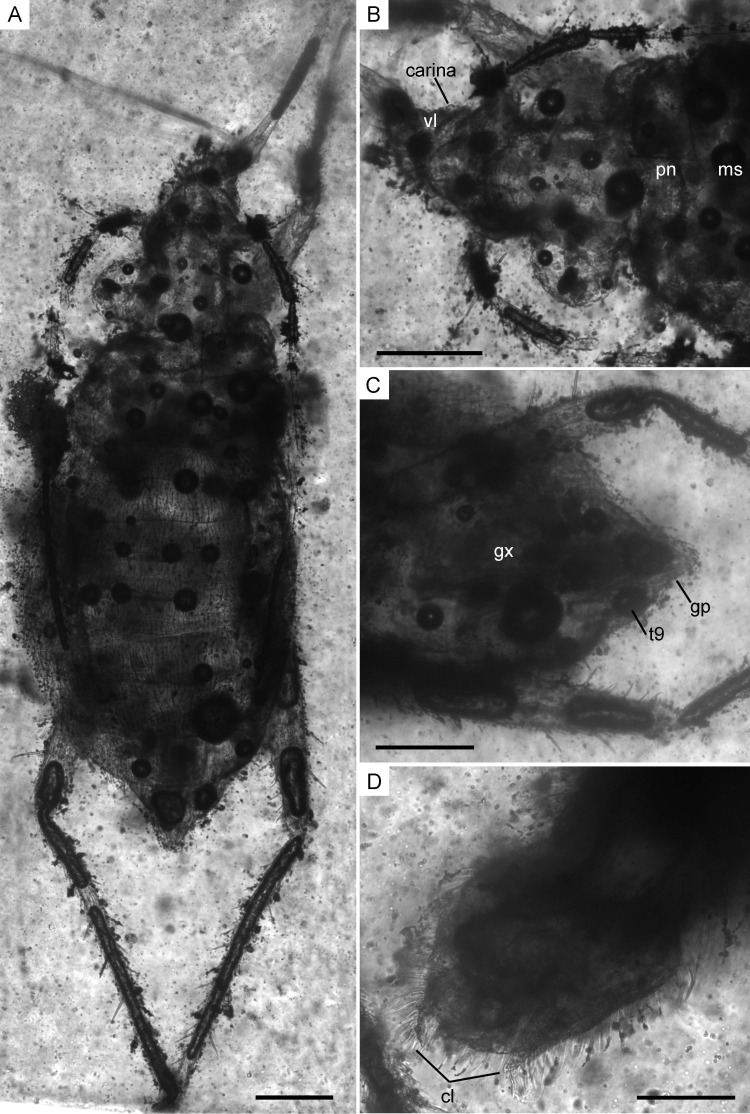
Infrared reflected photomicrographs of the holotype, female (MCNA 12806), and allotype, male (MCNA 12805), of *Glaesivelia pulcherrima* gen. et sp. n. (A) Dorsal habitus of holotype (MCNA 12806). (B) Dorsal close-up view of the head and anterior part of the pronotum. (C) Female genitalia of holotype (MCNA 12806) in the ventral view. (D) Male genitalia of allotype (MCNA 12805) in the ventral view. cl, clasper; gp, gonoplacs; gx, gonocoxa; ms, mesonotum; pn, pronotum; t9, tergum 9; vl, ventral lobe. Scale bars: 0.2 mm. Images combine consecutive photographs taken at successive focal planes.

**Figure 8:**
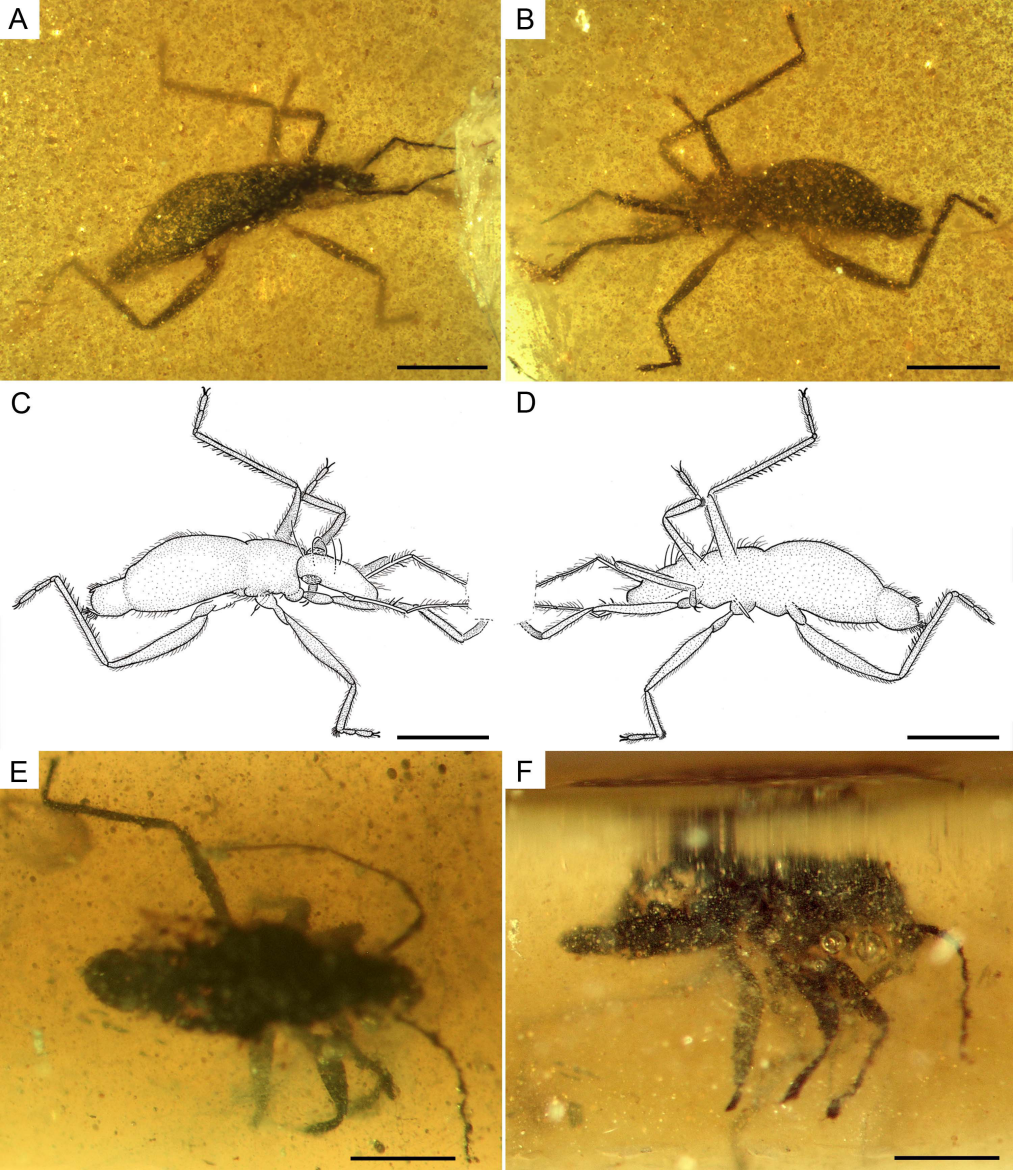
Photomicrographs and camera lucida drawings of the males of *Glaesivelia pulcherrima* gen. et sp. n (A) Dorso-lateral habitus of the allotype, male (MCNA 12805). (B) Ventro-lateral habitus of the allotype, male (MCNA 12805). (C) Drawing from A. (D) Drawing from B. (E) Dorsal habitus of MCNA 13326, male. (F) Lateral habitus of MCNA 13326, male. Scale bars: 0.5 mm. Images combine consecutive photographs taken at successive focal planes.

**Figure 9:**
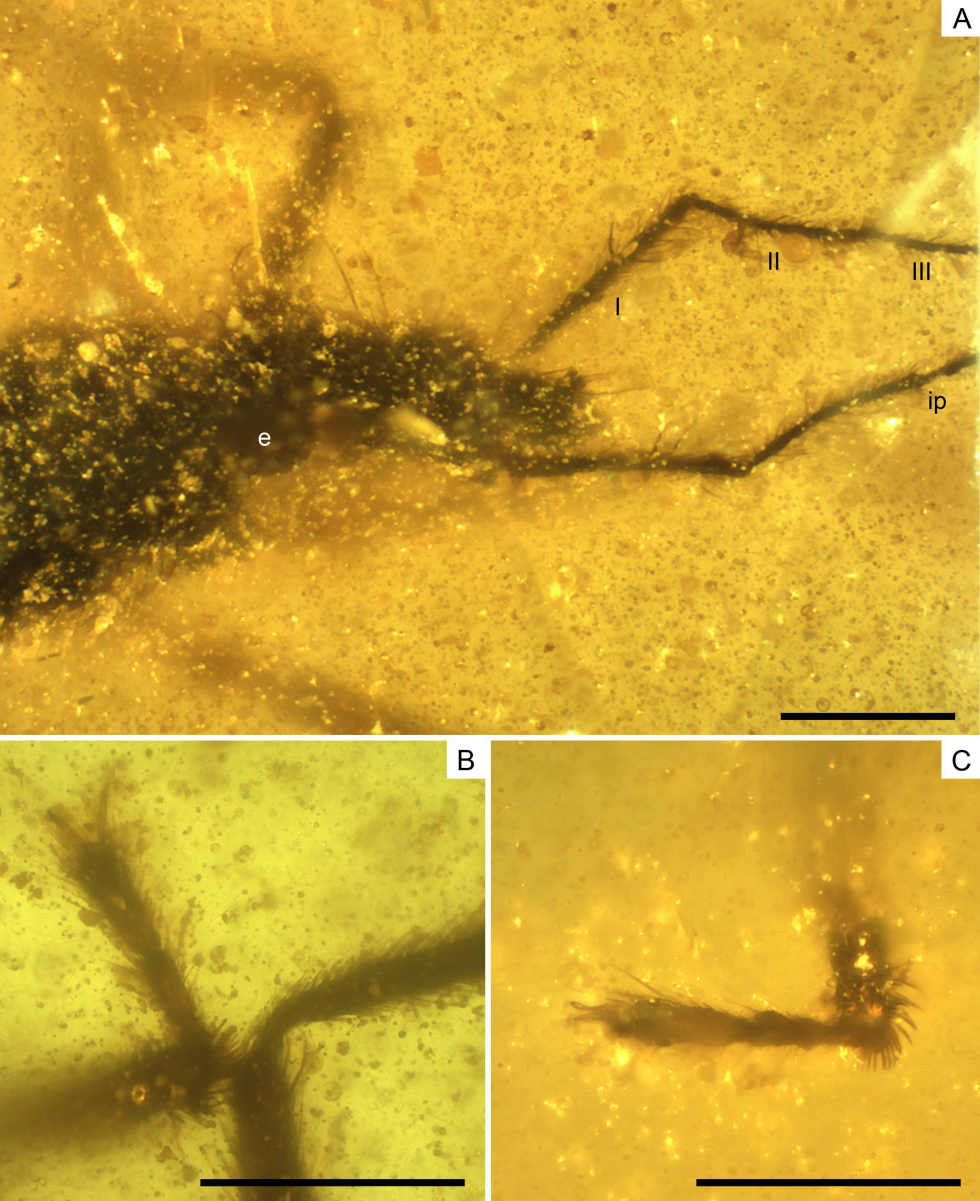
Photomicrographs of the allotype of *Glaesivelia pulcherrima* gen. et sp. n., male, MCNA 12805. (A) Dorso-lateral close-up view of the head and anterior part of the pronotum. (B) The left mesotarsus. (C) The right mesotarsus; note the mesotibial grooming comb. I, II, III, antennal segments I–III; e, eye; ip, internodial piece. Scale bars: 0.2 mm. Images combine consecutive photographs taken at successive focal planes.

**Figure 10:**
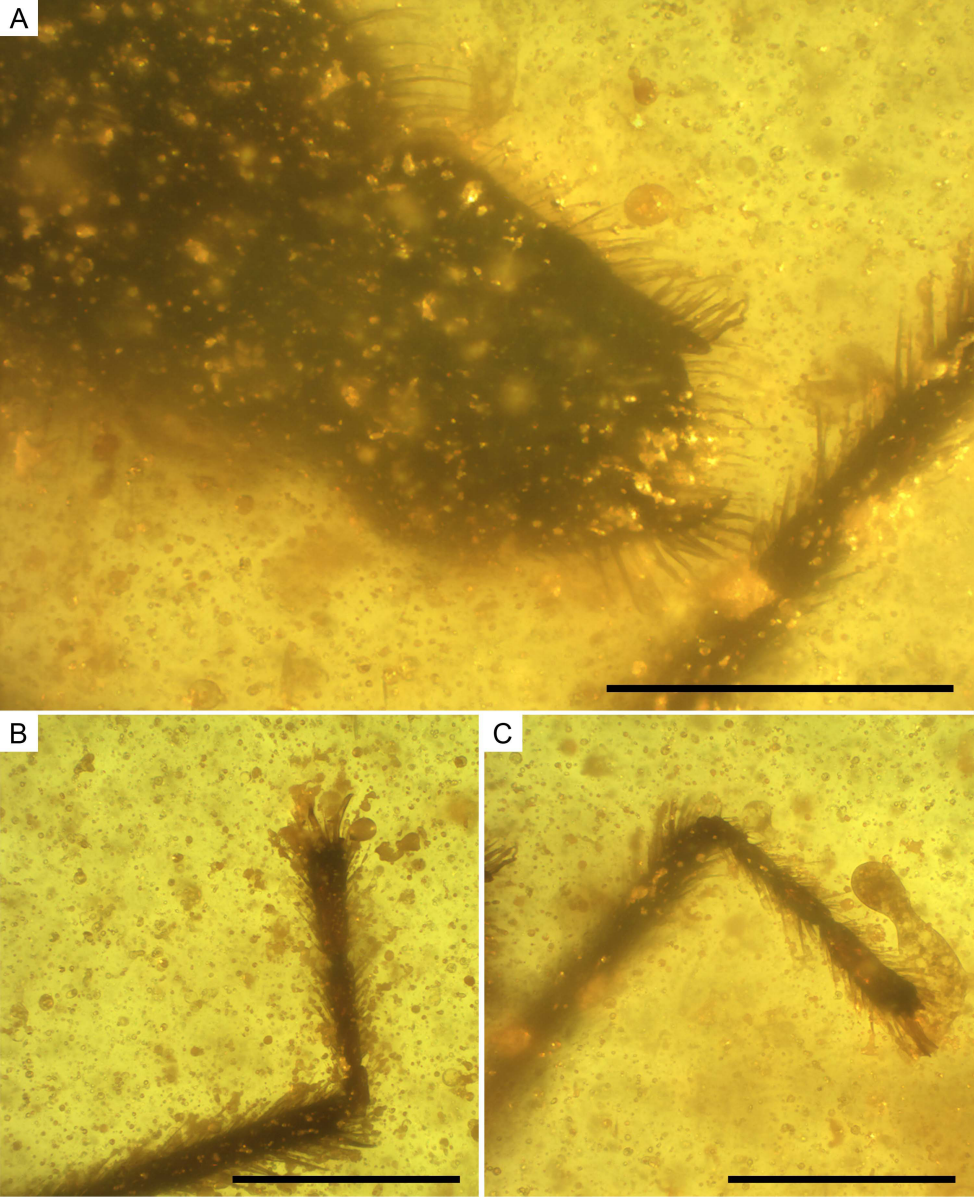
Photomicrographs of the allotype of *Glaesivelia pulcherrima* gen. et sp. n., male, MCNA 12805. (A) Male genitalia in the ventro-lateral view. (B) The left metatarsus. (C) The right metatarsus. Scale bars: 0.2 mm. Images combine consecutive photographs taken at successive focal planes.

*Glaesivelia pulcherrima* Sánchez-García & Solórzano Kraemer **sp. n.** urn:lsid:zoobank.org: act:64147FCE-2C91-4611-B62C-82858801C5AA

(Figs. 6–10, Table 1)

**Type material.** Holotype MCNA 12806, female, virtually complete, dorsally and laterally exposed. Preserved in dark yellow turbid amber, trimmed to 0.5 × 0.1 × 0.2 mm (in a trapezoid resin measuring 2.2 × 1.5 × 0.2 mm), which contains many impurities and bubbles obscuring some features of the specimen, especially the cuticular surface. The right mesothoracic leg is missing below the distal third of the tibia, while the metatarsi are lost at the surface of the amber. The entire head, rostrum, and even antennae are preserved, as are the thorax and abdomen (including genitalia).

Allotype MCNA 12805, male, virtually complete, dorsolaterally and ventrolaterally exposed. Preserved in dark yellow turbid amber, trimmed to 0.6 × 0.4 × 0.1 mm (in a trapezoid resin measuring 2.2 × 1.4 × 0.1 mm), containing many impurities and bubbles. Most of the antennae (missing from the base of the third segment) and right protarsus are lost at the surface of the amber. The left protarsal segments are not distinguishable due to preservation. Both type specimens were preserved as syninclusions with the holotype of *I. quisquilia* (MCNA 12804) and a *Microphorites* sp. (MCNA 12807).

**Age and locality.** Lower Cretaceous (Upper Albian); Peñacerrada I amber site (Peñacerrada I = Moraza), eastern area of the Basque-Cantabrian Basin, Burgos, northern Spain.

**Additional material.** MCNA 13326 (Figs. 8E–8F), male, poorly preserved in Lower Cretaceous Álava amber, Peñacerrada II amber site.

**Etymology.** The specific epithet *pulcherrima* is Latin for beautiful, and makes reference to the gorgeous habitus of the holotype.

**Diagnosis.** Same as for the genus (*vide supra*).

**Description of the holotype.** Female (Figs. 6, 7A–7C). Apterous form. Body (Figs. 6, 7A) suboval and rather stout, very small, length 1.61 mm, greatest width (across abdomen) 0.71 mm, length 2.27× the greatest width. Body surface and appendages covered with fine to coarse recumbent to semi-erect long setae.

Head (Fig. 7B) relatively long, not deflected, clearly extended in front of the eyes, length 0.29 mm, much longer than wide, greatest width (across eyes) 0.25 mm, with sides nearly parallel; subequal in length to the middorsal length of thorax and clearly narrower than pronotum; anterior part slightly declivent in side view; ventral lobes weakly-developed; ventral surface of head laterally bordered by ridged longitudinal carinae; anteclypeus with a pad of long erect setae. Three pairs of trichobothria on dorsal head surface, long, apparently not equally spaced in the longitudinal direction; the posterior pair is the longest and occurs towards the base of the head, just before the posterior margin of the eyes, while the other two pairs arise from swellings in the anterior part of the head, well in front of the eyes. No distinct median groove on head.

Compound eyes (Fig. 7B) spherical, large, diameter 0.17 mm, that are not touching and only slightly separated from the anterior margin of the pronotum, with more than 30 ommatidia; ocular setae not visible. Ocelli absent.

Antennae (Figs. 6, 7A) long, length 1.37 mm, just reaching the sixth abdominal segment when directed backwards, flagelliform, with segments 3–4 much longer and thinner than segments 1–2; antennal tubercles moderately prominent, slightly projected laterally, and situated near apex of head; first antennal segment slightly longer than second, lengths 0.22 and 0.17 mm, respectively (measurements possibly underestimated due to fossilization position), the first segment with three ante-apical spinous hairs; third and fourth segments subequal in length, lengths 0.48 and 0.50 mm, respectively, the fourth segment uniformly sclerotised throughout.

Rostrum (Figs. 6, 7A) long and slender, with apex reaching metacoxae; first labial segment slightly longer than second; third segment very long, 4.02× the length of fourth, lengths 0.64 and 0.16 mm respectively, the third segment swollen at base and clearly tapering towards the apex.

Thorax (Figs. 6C, 7B) not prolonged, length 0.28 mm, greatest width (across metanotum) 0.58 mm; pro-, meso-, and metanotum visible from above as transverse plates, with lateral margins rounded; pronotum without collar, longer than mesonotum, lengths 0.12 mm and 0.09, respectively; metanotum the shortest, length 0.06 mm; intersegmental suture between pro- and mesonotum slightly pointed in the middle; intersegmental suture between meso- and metanotum slightly curved; metanotum with posterior margin weakly curved. Metathoracic scent gland not visible.

Legs (Figs. 6B, 6D) inserted close to the ventral midline of the body, short, relatively robust, and with strong spinous hairs; metathoracic leg the longest; coxae relatively long; femora moderately incrassate in the middle, nearly twice as thick as the corresponding tibiae; metafemur the longest, not surpassing the abdominal apex; anterior margin of pro- and mesofemur with at least one and two ante-apical spinous hairs, respectively, metafemur with at least two ante-apical spinous hairs on the outer margin and at least seven on the inner margin; tibiae slender; metatibia the longest, covered with scattered spinous hairs and up to four spinous hairs distally; lengths of profemur and protibia: 0.44 and 0.44 mm; mesofemur and mesotibia: 0.51 and 0.38 mm (measurements underestimated due to fossilization position); metafemur and metatibia: 0.56 and 0.76 mm; tarsi with three segments (not preserved at both metathoracic legs and the right mesothoracic leg); tarsal segments increasing in length from first to third segment in pro- and mesotarsus; basitarsomere of all legs very short and subcylindrical; lengths of protarsal segments 1–3: 0.03, 0.07, and 0.09 mm; mesotarsal segments 1–3 (measurements underestimated due to fossilization position): 0.02, 0.07, and 0.08 mm; claws simple, slender, and inserted apically into the distal tarsal segment; arolia not visible.

Abdomen (Figs. 6, 7A) long, length 1.04 mm, greatest width 0.71 mm, clearly widened at the middle, with broadly concave sides; mediotergites tapering in width towards the abdominal apex. Abdominal scent gland not visible. Genital segments (Fig. 7C) large and clearly protruding from pregenital abdomen; gonocoxae large and plate-shaped; gonapophyses elongate and laciniate; tergum 9 well developed; proctiger rounded; gonoplacs small and triangular in shape.

Macropterous adult form unknown.

**Description of the allotype.** Male (Figs. 7D, 8A–8D, 9, 10). Apterous form, very similar to female, but somewhat shorter (Figs. 8A–8D), length 1.56 mm, width measurements not available due to fossilization position. Other differences with the female are listed below.

Head (Fig. 9A) length 0.32 mm and longer than thorax. Three pairs of long trichobothria on dorsal head surface, their exact distribution obscured by preservation. Eyes (Fig. 9A) diameter 0.11 mm. Both antennae (Fig. 9A) polished off at base of third segment, with segment 3 much thinner than segments 1–2; first antennal segment subequal to or slightly longer than second, lengths 0.28 and 0.24 mm, respectively, the first segment with two ante-apical spinous hairs.

Thorax (Figs. 8A–8D) length 0.28 mm; pro-, meso-, and metanotum only visible in side view, thus impeding measurements; pronotum longer than mesonotum; intersegmental sutures poorly visible, the posterior margin of metanotum apparently curved. Legs (Figs. 8A–8D) with strong spinous hairs (although with a different pattern to that in the female, see below); lengths of profemur and protibia: 0.46 and 0.46 mm; mesofemur and mesotibia: 0.51 and 0.28 mm (measurements underestimated due to fossilization position); metafemur and metatibia: 0.55 and 0.65 mm; femora without strong spinous hairs; mesotibia with a grooming comb apically (Figs. 9B, 9C); metatibia with a row of spinous hairs along the inner margin; tarsi with three segments (not distinguishable in protarsus due to preservation); tarsal segments increasing in length from first to third segment (Figs. 9B, 9C) except in metatarsus (Figs. 10B, 10C) (second metatarsal segment subequal in length to the third); lengths of mesotarsal segments 1–3: 0.05, 0.06, and 0.09 mm; metatarsal segments 1–3: 0.04, 0.10, and 0.10 mm.

Abdomen (Figs. 8A–8D) length 0.97 mm. Genital segments (Figs. 7D, 10A) large and clearly protruding from pregenital abdomen; proctiger very prominent, distally widened, length 0.24 mm, width 0.20 mm; claspers large and slender, symmetrical, shallow sickle-shaped, with the blade slightly curved and narrowing apically and displaying a series of thick setae; each clasper placed in the lateral margin of the pygophore.

**Observational notes.** Two morphotypes representing a male and female of the same species belonging to the rare family Mesoveliidae were observed in the same piece of amber together with the holotype female of *Iberovelia*. Morphological details of both the male and female genitalia of *Glaesivelia* were assessed using infrared microscopy. Male genital segments are often more conspicuous than female ones, which might be concealed or even retracted into the pregenital abdomen. Some important characters of the *Glaesivelia* male genitalia have not been included in the diagnosis of the new genus, but in the description because they are unknown in *Iberovelia*. However, these characters are remarkably different from those observed in all other males belonging to extant genera and are thus of potential diagnostic significance. One of these characters is the well-developed sickle-shaped male claspers of *Glaesivelia*, which probably had a functional role during copulation.

Extant mesoveliids often exhibit secondary sexual dimorphism in body size (males are usually shorter), certain body proportions (e.g., the relative lengths of the thoracic segments), and in the presence/absence of spines and their distribution. In this regard, the *Glaesivelia* allotype differs from the female holotype by the slightly smaller size and the different ratio of head to thorax lengths. Andersen & Polhemus (1980) reported similar observations in Recent *Cryptovelia*, with the length of the head being subequal to the middorsal length of the pro- and mesonotum in males and the length of the whole thorax in females. Other minor differences between *Glaesivelia* males and females include the distribution of the spines on the legs and antennae, as well as the presence of a comb of modified macrotrichia on the apexes of male mesotibiae.

Regarding wing dimorphism, most Recent Mesoveliinae are only known from apterous specimens, such as the fossil genera described herein. *Madeovelia* Poisson, 1959 and *Mesoveloidea* Hungerford, 1929 are the only known monomorphic macropterous specimens, while some *Mesovelia* species have been described as wing-dimorphic comprising both macropterous and apterous forms (Damgaard et al., 2012).

**Infrared microscopy.** The severely darkened cuticle of the specimens, hidden behind the occluded amber, rendered it impossible to resolve some detailed characters with light microscopy. Therefore, some details of the head, such as the ventral lobules and lateral carinae, the segmentation of the thorax and abdomen, the density of body setae, and the female and male genital structures were examined using infrared microscopy (Fig. 7). Infrared microscopy is still not widely applied in the study of inclusions in amber; however, in accordance with previous studies, our results show that this technique is promising and a good alternative to the systematic study of organisms preserved in amber that are not clearly visible.

## Discussion

The new genera *Iberovelia* and *Glaesivelia* can be assigned to Gerromorpha based on the presence of large rounded compound eyes, a head that is not constricted transversely, a body covered with a distinct pile of microsetae, and the presence of three pairs of cephalic trichobothria inserted into deep cuticular pits (Andersen, 1982; Schuh & Slater, 1995).

The monophyletic status of the Mesoveliidae is supported by six characters, according to Andersen (1982): (1) an obliquely truncated anterior end of the egg; (2) a circular eclosion split of the egg shell, an absent embryonic egg buster, and an eclosion by means of an embryonic bladder; (3) absent dorsal indentations and apodemes of the head; (4) reduced forewing venation (wings with only three closed cells); (5) the first abdominal mediotergite of the macropterous adult form displaying a pair of longitudinal ridges; and (6) a specialised ejaculatory bulb and pump in the male genital tract. Damgaard (2008b) diagnosed Mesoveliidae on the presence of an ejaculatory bulb and pump in the male genital tract and an absent embryonic egg burster. Although none of these characters were available from our fossils, the presence of a well-developed ovipositor along with the three pairs of cephalic trichobothria indicated that the new genera belonged to Mesoveliidae (Damgaard et al., 2012). A well-developed female ovipositor is a plesiomorphic state in Heteroptera. Most Gerromorpha families lack this character, thus restricting the assignment of the new taxa to the family Mesoveliidae or to the subfamily Rhagadotarsinae (Gerridae), in which the enlarged ovipositor has probably been achieved secondarily (Damgaard, 2008a). However, Rhagadotarsinae members have a very distinct appearance (Andersen, 1982) compared to mesoveliids and can therefore be easily distinguished.

Moreover, the two new genera described here also have features typical of the majority of apterous forms of mesoveliid genera, namely a porrect head extending in front of the eyes, absent ocelli, a thorax divided into three simple segments with no differentiation of a scutellum or wing pads, a mesonotum medially subequal to or shorter than the pronotum, three-segmented tarsi, and claws inserted apically into the distal tarsomere (Polhemus & Chapman, 1979; Andersen, 1982; Damgaard et al., 2012).

Pondweed bugs have retained a number of plesiomorphic characteristics and have a rather generalised heteropteran appearance (Damgaard et al., 2012). This, together with the limited number of external diagnostic features for family assignment, leads to any assignment of fossils to the family being potentially dubious. To date, only the Jurassic *Gallomesovelia grioti* from marine limestones of the area around Orbagnoux (Rhône, France) (Nel et al., 2014) and the Cretaceous *Emilianovelia audax* and *Malenavelia videris* from French amber (Solórzano Kraemer et al., 2014) plus an immature gerromorphan described by Perrichot, Nel & Néraudeau (2005), have been assigned to Mesoveliidae, while the Miocene *Mesovelia dominicana* remains the only Cenozoic mesoveliid described (Garrouste & Nel, 2010). All the other taxa previously attributed to Mesoveliidae are currently considered Heteroptera *incertae sedis* (Damgaard et al., 2012).

### Subfamily assignment

Andersen (1999) and Andersen & Polhemus (2003) investigated the phylogenetic relationships among extant Mesoveliidae genera, describing three major clades: (1) *Madeovelia* and *Mesoveloidea* in the subfamily Madeoveliinae; (2) *Mesovelia* Mulsant & Rey, 1852, *Speovelia* Esaki, 1929, and *Cavaticovelia* Andersen & Polhemus, 1980; and (3) *Phrynovelia* Horváth, 1915, *Cryptovelia* Andersen & Polhemus, 1980, *Darwinivelia* Andersen & Polhemus, 1980, *Mniovelia* Andersen & Polhemus, 1980, *Austrovelia* Malipatil & Monteith, 1983, and *Nereivelia* Polhemus & Polhemus, 1989 in the subfamily Mesoveliinae Douglas & Scott, 1867. The genus *Seychellovelia* Andersen & Polhemus, 2003 was later described and included in clade 3 (Andersen & Polhemus, 2003).

Damgaard et al. (2012) showed that the current classification of mesoveliid subfamilies and genera require revision, identifying *Mesoveloidea*, from the subfamily Madeoveliinae, as a sister group to *Mniovelia*, from Mesoveliinae, thus making the latter subfamily paraphyletic. Moreover, the genus *Mesovelia* was also shown to be paraphyletic, since several species were identified as sister groups to the genus *Phrynovelia* or *Speovelia*. Due to the high percentage of monotypic or species-poor genera, the limited number of diagnostic character combinations used for describing taxa, and the many clades diagnosed on putative convergences or homoplasies, Damgaard et al. (2012) rejected the subfamily classification. However, we have employed the clades in Andersen (1999) for clarity.

The placement of *Glaesivelia* and *Iberovelia* close to the extant genera *Madeovelia* and *Mesoveloidea* or the fossil genus *Gallomesovelia* (currently in Madeoveliinae) is clearly unsupported given the overall shape of the head (deflected in front of the eyes instead of extended in *Glaesivelia* and *Iberovelia*), the occurrence of winged adults (instead of apterous in *Glaesivelia* and *Iberovelia*), and the preapical insertion of claws (instead of apical in *Glaesivelia* and *Iberovelia*). Both apical pretarsal structures and extended heads are shared with other gerromorphan families, probably corresponding to plesiomorphic states.

The monophyly of the subfamily Mesoveliinae is supported by the shared apomorphic characteristics of an ejaculatory bulb of the male with a broad pump flange, the anterior end of the egg with a complete pseudopercular rim, and the tergal and stemopleural parts of the prothorax not usually being delimited by sutures (Andersen, 1999). We have refrained from formally including the genera described here in Mesoveliinae, as the listed characters are not known in our fossils and the diagnostic character combinations used for describing Mesoveliidae taxa are currently under revision. Table 1 shows the characters shared with some genera currently in Mesoveliinae.

The new fossil genera *Glaesivelia* and *Iberovelia* are most similar to the genera included in clade 3 through their flagelliform antennae (two distal segments much thinner and longer than the two basal segments and usually setose), a not simple ventral head, a mesonotum subequal to or shorter than the pronotum, and the relatively small gonoplacs of the female genitalia (except for the subflagelliform antennae and the large gonoplacs of *Darwinivelia*). *Glaesivelia* also shares with these genera (except *Mniovelia*) a head subequal in length to or longer than the thorax. Conversely, *Mniovelia* and the other new fossil genus *Iberovelia* share a head that is shorter than the thorax, although the difference in length is very small in *Iberovelia*. It has also been shown some variability in the relative lengths of the head and thorax in *Cryptovelia*, with *C. terrestris* males having a head that is shorter than the thorax (Table 1). As for the relative lengths of the metatarsal segments, the second segment is never longer than the third in *Glaesivelia*, as seen in almost all the genera in clade 3, whereas the second tarsal segment is longer than the third in the fossil *Iberovelia* as well as in *Mesovelia*, *Speovelia* and *Cavaticovelia* (clade 2), and *Mniovelia* (clade 3). Furthermore, *Cryptovelia stysi* males have a longer second metatarsal segment compared to the third. *Glaesivelia* and *Iberovelia* also share with most genera the spherical and large compound eyes. However, a trend towards smaller eyes has been observed in some species inhabiting caves or other secluded places (e.g., *Austrovelia caledonica* Malipatil & Monteith, 1983 and *Darwinivelia fosteri* Andersen & Polhemus, 1980), while the eyes are vestigial in the leaf litter-inhabiting *Cryptovelia* species. Remarkably, the two new fossil genera differ from all the genera in clade 3 by having strong antennal and femoral spines, as found in *Mesovelia*, *Speovelia* and *Cavaticovelia* (clade 2). Although antennal spines are not present in the clade 3 genera, some of them present a variable number of bristles in the first segment that are distinct from the surrounding setae (one in *Seychellovelia*, three in *Cryptovelia*, and two in *Mniovelia*). Similarly, thin femoral spines are present in *Darwinivelia*, while strong spines are either absent or present in *Cryptovelia*. Table 1 presents the other characters with taxonomic significance. However, there are additional features that can be used to easily distinguish the new fossil taxa from the Recent genera, such as the absence of the very short second antennal segment and small rounded male claspers diagnostic of *Cryptovelia* (male claspers are well developed and sickle-shaped in *Glaesivelia*), the absence of the tripartite first abdominal tergum of *Phrynovelia*, or the absence of a deep glabrous median furrow on the vertex of the head and the very large protruding eyes of *Mniovelia*.

The general habitus of our fossils is very close to those of the two fossil genera *Emilianovelia* and *Malenavelia*, currently assigned to Mesoveliinae (Solórzano Kraemer et al., 2014). They share relatively small bodies (around 1.4 mm long in *Emilianovelia* to 1.6 mm long in the other taxa) with moderately long heads and appendages, normal-sized eyes (not reduced), and flagelliform antennae. *Emilianovelia* and *Malenavelia* were originally described as having subflagelliform antennae, but it is now clear that the antennae are flagelliform. *Iberovelia* shares with *Emilianovelia* the following diagnostic characters, as listed in Solórzano Kraemer et al. (2014): a rostrum reaching the metacoxae; a pronotum longer than the mesonotum; and an anteclypeus with a pad of long erect hairs. We can add that they also share very long antennae surpassing the length of the body, the presence of two ante-apical spinous hairs on the first antennal segment, long metafemora reaching the abdominal apex, and a longer second metatarsal segment compared to the third segment (ca. twice the length). However, *Iberovelia* differs from *Emilianovelia* through some body proportions, its slightly declivent head (instead of abruptly declivent in *Emilianovelia*), different distributions of spinae on the femora (1:1:1 from pro- to metafemora in *Emilianovelia* and 1:2:1 in *Iberovelia*) and metatibiae (only a few spinous hairs on the inner margin and a series of spinous hairs on the outer margin in *Iberovelia*; and a few spinous hairs on the outer margin and a series of spinous hairs on the inner margin in *Emilianovelia* after Solórzano Kraemer et al., 2014, fig. 1), and the small gonoplacs (instead of large and elongate in *Emilianovelia*).

*Glaesivelia* shares with *Malenavelia* the following diagnostic characters (Solórzano Kraemer et al., 2014): a rostrum reaching the metacoxae; a pronotum longer than the mesonotum; a metanotum with a slightly curved posterior margin; and short metafemora not surpassing the abdominal apex. However, *Glaesivelia* has a slightly declivent head (instead of the abruptly declivent in *Malenavelia*), weakly developed ventral lobes (instead of the well-developed lobes in *Malenavelia*), and a venter of the head possessing a ridged longitudinal carina (instead of a simple one in *Malenavelia*). Other characters shared by *Glaesivelia* and *Malenavelia* include the short antennae not reaching the length of the body, the second metatarsal segment being as long as or slightly longer than the third segment (after Solórzano Kraemer et al. (2014), Fig. 3C and Fig. 4D), and the small gonoplacs. The male genitalia of *Glaesivelia* is clearly larger than that of *Emilianovelia* and *Malenavelia*, and has well-developed claspers. Although the male genitalia of the *Emilianovelia* holotype was described as having a pair of lateral spinous extensions, these are clearly smaller than those in *Glaesivelia* and it is unclear whether they correspond to a clasper. It should be noted that although the lack of spines in *Malenavelia* is likely to be an artefact due to scanning resolution, this, however, prevents its comparison with *Glaesivelia*.

### Palaeoecology

Modern mesoveliids live in moist surroundings such as humid terrestrial (litter and moss) or marginal aquatic habitats, or on water surfaces extensively covered with floating leaves of water plants (Andersen & Weir, 2004). Humid terrestrial habitats (hygropetric) are not necessarily close to free water, consisting of different types of solid substrates covered by a thin film of water (Andersen, 1982; Andersen, 1998). The cosmopolitan genus *Mesovelia* contains both hygropetric species and surface-inhabiting species living on plant-covered water surfaces (Damgaard et al., 2012). However, mesoveliids are most frequently found in humid terrestrial environments, with some genera and species even occurring at a great distance from the nearest body of water (Andersen & Polhemus, 1980; Damgaard et al., 2012). The genera *Phrynovelia* (New Caledonia, New Guinea, and the Philippines) and *Cryptovelia* (Brazil and Borneo) inhabit soil and leaf litter in temperate and intertropical rain forests (Horváth, 1915; Andersen & Polhemus, 1980; Malipatil & Monteith, 1983), while *Mniovelia* (New Zealand) and *Seychellovelia* (Seychelles) live in moist terrestrial environments among mosses and litter on the floor of rain forests on misty mountains far from ponds or streams (Andersen & Polhemus, 1980; Andersen & Polhemus, 2003). The dependence on water varies. *Austrovelia* inhabits ground litter in rain forests in Australia and New Caledonia, but reproduces only in the wet season when water-filled leaves among the litter provide a habitat for early stages (Malipatil & Monteith, 1983). Moreover, there are two genera of Mesoveliinae—*Speovelia* (Japan and Mexico) and *Darwinivelia* (Galapagos Islands and Brazil)—that occur in narrow crevices and holes in an intertidal marine environment (Andersen & Polhemus, 1980), while *Nereivelia* and other members of *Darwinivelia* are found on mangrove flats (Polhemus & Polhemus, 1989) and the troglobitic *Cavaticovelia aaa* frequents lava tubes in Hawaii (Andersen & Polhemus, 1980).

There are no definite associations within Mesoveliidae between the physical nature of the environment and the diagnostic characters of the species inhabiting different types of habitats (from humid terrestrial to freshwater to marine). However, studies on aquatic Heteroptera have shown some variability in the structure and arrangement of setae (Perez-Goodwyn, 2009), with the highest densities of microthrichia occurring in marine taxa. Despite the descriptions not being usually precise regarding this feature, some typically humid terrestrial mesoveliid genera, such as *Phrynovelia*, *Cryptovelia*, *Mniovelia*, and *Cavaticovelia*, have been described as having glabrous abdominal sternites or sternopleuron. In *Glaesivelia* and *Iberovelia*, the body is covered with long setae that are not very dense, but as far it is possible to observe, do not leave any parts exposed (Fig. 7). It is also important to note the presence of a distal comb of modified macrotrichia on the mesotibiae of *Glaesivelia* males (Fig. 9C). Grooming is an important activity in semi-aquatic bugs, which use specialised structures to keep the hair layers of the legs and body tidy and free of dust and water drops (Andersen, 1982).

**Figure 11:**
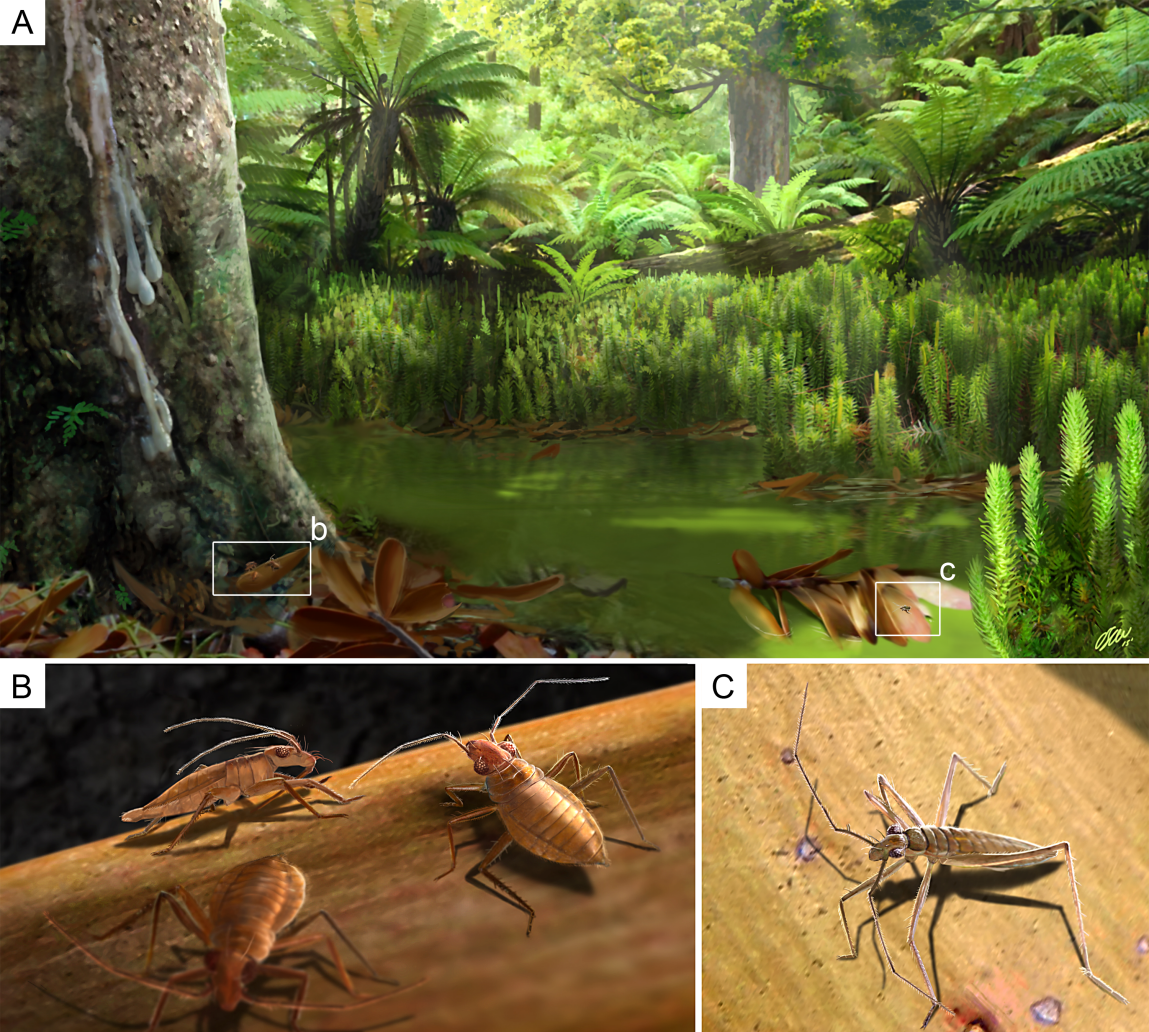
Palaeoecological reconstruction of *Glaesivelia pulcherrima* gen. et sp. n. and *Iberovelia quisquilia* gen. et sp. n. (A) Hypothetical swamp environment of the Cretaceous (Albian) Spanish amber forest (*sensu* Peñalver & Delclòs, 2010). (B) Aggregative behaviour of *G. pulcherrima*; male (specimen in side view) and two females. (C) Reconstructed habitus of *I. quisquilia* female. Illustrations by O Sanisidro with scientific supervision.

Thus, the fossil mesoveliids described herein probably lived in a humid terrestrial habitat (hygropetric), with entrapment in resin likely occurring out of water (Fig. 11). Andersen & Polhemus (1980) suggested that the most ancestral habitat of Mesoveliidae was ‘humid terrestrial and/or marginal aquatic including litter or wet soil, watersoaked, moss, and seeping rock faces’. This is consistent with the results in Damgaard et al. (2012), in which the terrestrial *Austrovelia* resolves as the sister group to all the other genera and could actually represent the original life style of the whole family. However, the preferred habitat of the Spanish fossil mesoveliids might also be characterized as marginal aquatic and therefore transitional between terrestrial and freshwater environments (Fig. 11). Indeed, the rest of the arthropod assemblage is consistent with such an environment, with numerous representatives of the ground habitat of humid forests preserved in ‘litter amber’ (Sánchez-García et al., 2015; Sánchez-García, Arillo & Nel, 2016; Arillo, Subías & Sánchez-García, 2016; Sánchez-García & Engel, 2016a; Sánchez-García & Engel, 2016b).

In this regard, the extreme scarcity of Mesozoic Mesoveliidae fossil records could be related to the confined microhabitats of primitive mesoveliids, which made their capture in tree resins unlikely. After actualistic experiments in a tropical forest in Chiapas (Mexico), Solórzano Kraemer et al. (2015) posed that the underrepresentation of several groups could be explained by their preferred habitats in leaf litter. The presence of mesoveliids in the arthropod fauna of Cretaceous Spanish amber strongly supports this hypothesis, as it probably stems from the unusual preservation of litter-inhabiting species in addition to organisms living on trees (Sánchez-García et al., 2015). More importantly, the discovery of three specimens in the same piece of amber indicates possible aggregative behaviour, thereby representing the earliest occurrence of such ethology for Mesoveliidae. Similarly, three and four mesoveliids, including males and females, have been reported fossilised in two pieces from Cretaceous French amber (Solórzano Kraemer et al., 2014). Naturally, there are many taphonomic factors that might cause individuals to be preserved together and that do not relate to a behavioural phenomenon. However, given the scarce fossil record of these bugs, it seems particularly unlikely that several specimens were repeatedly captured in the same small piece of amber due to chance alone, supporting the idea that the three specimens were in close association when engulfed in a resin flow very close to their habitat. Indeed, gregarious behaviour is common among Recent semi-aquatic bugs (Andersen, 1982).

## Conclusions

The relative significance of morphological and molecular characters in mesoveliid phylogeny is presently not well understood. The criteria for distinguishing supra-specific taxa (some genera and subfamilies) are often not well defined, increasing the difficulty of defining and assigning new taxa. Some morphological characters have evolved more rapidly in response to environmental or ecological (lifestyle) selection pressures (e.g., body shape and size, eye development, and leg morphology), while others have been less influenced and give a better phylogenetic signal. The many convergences associated with adaptation to a cryptic life and the reductions of some features (e.g., wings) have resulted in the few convincing synapomorphies available. This problem is evident when new fossil species are discovered (even three-dimensional and well-preserved species such as those described herein) and are assigned to the rather generalised family Mesoveliidae based on the few applicable characters.

Interpretation of the diversity and geographical distribution relies on understanding the phylogenetic relationships between species, and therefore between genera and even higher taxa. Marked advances in mesoveliid phylogeny have been made in recent years (Damgaard, 2008a; Damgaard, 2008b; Damgaard et al., 2012), but further work is still required to establish a robust phylogeny of the group.

The finding of two new fossil mesoveliid genera from Spanish amber considerably expands the taxonomic records of the family. It is also remarkable that such fossils probably represent the earliest mesoveliinae described to date and one of very few known amber inclusions of the family. Nevertheless, it is evident from our current knowledge of fossil mesoveliids that they were a diverse group during the Cretaceous and of considerable ecological significance in certain moist Mesozoic terrestrial habitats. Given that Spanish amber appears to preserve a large amount of litter fauna, sampling considerable material from the forest floor, it is hoped that further mesoveliid material will come to light.

## Additional Information and Declarations

### Data Availability

The following information was supplied regarding data availability: The measurements in Table 1 are the raw data. The material described herein is housed in the Museo de Ciencias Naturales de Álava (MCNA), Vitoria-Gasteiz (Álava, Spain).

### New Species Registration

The following information was supplied regarding the registration of a newly described species: Publication LSID: urn:lsid:zoobank.org:pub:5EBBDE3B-9A12-4476-BC96-776651350175 *Iberovelia* Sánchez-García & Nel gen. n. urn:lsid:zoobank.org:act:9093AD2B-3AFC-4345-B35B-08139856ECFA *Iberovelia quisquilia* Sánchez-García & Nel sp. n. urn:lsid:zoobank.org:pub:5EBBDE3B-9A12-4476-BC96-776651350175 *Glaesivelia* Sánchez-García & Solórzano Kraemer gen. n. urn:lsid:zoobank.org:act: DAC68787-5F0A-462A-BCBB-2CDD1E6991D6 *Glaesivelia pulcherrima* Sánchez-García & Solórzano Kraemer sp. n. urn:lsid:zoobank.org:act:64147FCE-2C91-4611-B62C-82858801C5AA.
